# Rapid On-Site Evaluation Does Not Improve Endoscopic Ultrasound-Guided Fine Needle Aspiration Adequacy in Pancreatic Masses: A Meta-Analysis and Systematic Review

**DOI:** 10.1371/journal.pone.0163056

**Published:** 2016-09-22

**Authors:** Fanyang Kong, Jianwei Zhu, Xiangyu Kong, Tao Sun, Xuan Deng, Yiqi Du, Zhaoshen Li

**Affiliations:** 1 Department of Gastroenterology, Changhai Hospital, Second Military Medical University, Shanghai, China; 2 Shanghai Medical College of Fudan University, Shanghai, China; Universita degli Studi di Napoli Federico II, ITALY

## Abstract

**Background and Objectives:**

Rapid on-site evaluation (ROSE) during endoscopic ultrasonography-guided fine needle aspiration (EUS–FNA) of pancreatic masses has been reported to be associated with improved adequacy and diagnostic yield. However, recent observational data on the impact of ROSE have reported conflicting results. A meta-analysis and systematic review was therefore conducted to evaluate the contribution of ROSE during EUS-FNA of pancreatic masses.

**Method:**

A systematic search was conducted in MEDLINE/Pubmed and EMBASE databases for studies comparing the efficacy of ROSE between patients in two cohorts. Outcomes considered included diagnostic adequate rate, diagnostic yield, number of needle passes, pooled sensitivity and specificity. Findings from a random-effects model were expressed as pooled risk difference (RD) with 95% confidence intervals (CIs).

**Results:**

A total of 7 studies (1299 patients) was finally included and further analyzed in the current meta-analysis. EUS-FNA with ROSE could not improve diagnostic adequacy (RD = 0.05, 95% CI: -0.01–0.11) and diagnostic yield (RD = 0.04 95%CI: -0.05, 0.13). The number of needle passes showed no statistically significant difference with and without ROSE (RD = -0.68 95%CI: -2.35, 0.98). The pooled sensitivity and specificity of ROSE group were 0.91 (95%CI: 0.87, 0.94) and 1 (95%CI: 0.94, 1.00). The pooled sensitivity and specificity of non-ROSE group were 0.85 (95%CI: 0.80, 0.89) and 1 (95%CI: 0.95, 1.00). ROSE group and non-ROSE group showed comparable sensitivity and specificity.

**Conclusion:**

Compared to historical reports of its clinical efficacy in patients with pancreatic lesions, ROSE may be not associated with an improvement of diagnostic yield, adequate rate, pooled sensitivity and specificity.

## Introduction

Pancreatic cancer is still one of the most lethal diseases and fourth leading cause of cancer related deaths [1,2]. Attributed to the aggressive biology, pancreatic cancer is diagnosed at an unresectable stage in most cases. Around 15–20% of patients have resectable disease, but less than 20% survival to 5 years[1]. Differential diagnosis of pancreatic cancer is considered to be a frequent clinical challenge. Retroperitoneal location of the pancreas also obscures the diagnosis of early pancreatic cancer. Therapeutic strategy in this context mainly relied on the determination or exclusion of malignancy. Therefore, a pathological diagnosis is the most valuable reference to make an optimal therapeutic decision.

Endoscopic ultrasound (EUS) is a well-established modality to diagnose and stage a variety of gastrointestinal and nongastrointestinal disorders, including pancreatic cancer[3]. EUS-guided fine needle aspiration (EUS-FNA) emerged as a minimally invasive technique to obtain cytological specimens. Accuracy of EUS-FNA has been evaluated in several studies in pancreatic cancer patients ranging from 60% to 96% [3–6]. By enhancing diagnostic sensitivity, staging accuracy, and prognostic determination [4,7], EUS-FNA helps guide patient care and improve outcomes.

ROSE is performed by an assessor (cytopathologist, cytotechnologist) to give an immediate evaluation for adequacy of sampling, thereby increasing the accuracy for obtaining a definitive diagnosis and reducing the need for additional needle passes. Recently, rapid on-site evaluation (ROSE) has been reported to be associated with a 10–15% rise of diagnostic yield in the EUS performance. Good agreement with the final cytopathological diagnosis is provided by the implementation of ROSE [8–11]. Meta-analyses revealed that on-site cytopathology evaluation could improve diagnostic adequacy and accuracy for malignancy detection [12–14]. However, new and high quality studies published recently reported convertible conclusions [15,16]. Design inadequacies of previous meta-analysis limited their capacity in completely addressing this clinical question and left the benefits of ROSE remained controversial. No significant benefit would be brought with the presence of a cytopathologist. We therefore aim to conduct a systematic review and meta-analysis on the efficacy of ROSE for patients undergoing EUS–FNA of pancreatic masses.

## Methods

This meta-analysis was conducted according to the guidelines of the preferred reporting items for systematic reviews and meta-analysis (PRISMA) statement (S1 PRISMA Checklist).

### Literature search

Medline/Pubmed and EMBASE were systematically searched by two authors (K.F. and Z.J.) to identify studies that compared the performance of EUS-FNA with and without ROSE for sampling the pancreatic masses. The key search words were “on-site,” “endoscopic ultrasound,” “cytopathologist/cytopathologic,” “cytotechnologist,” “fine needle aspiration,” “rapid evaluation” and “pancreatic”. No restrictions were placed on the study dates and language. The literature search was made from the beginning of indexing for each database to 5 March 2016. The identified studies were initially screened for eligibility by two authors (K.F. and Z.J.) independently. with no restriction in language. Any disagreement between the reviewers was resolved by the third author (K.X.).

### Inclusion criteria

All the eligible studies should satisfy the following inclusion criteria: 1) comparison in two cohorts of patients (with ROSE vs. without ROSE); 2) clinical trials targeting pancreatic lesions; 3) At least one of the following outcomes measures: diagnostic adequacy, diagnostic yield, number of needle passes required for diagnosis, diagnostic characteristics (number of true-positive, true-negative, false-positive, and false-negative observations). For studies with multiple publications from the same population source, only data from the most recent publication was included.

### Exclusion criteria

We excluded studies based on the exclusion criteria: 1) Study published in abstract format; 2) Detailed information unable to be extracted.

### Data extraction

The following information from the selected studies was independently extracted and assimilated by two of the authors (K.F. and Z.J.). Any disagreement between the two authors was resolved following consultation with the third author (K.X.).The following data were extracted from each study: design type of study, number of study centers, location, patient demographics, lesion characteristics, needle features, procedure details, diagnostic characteristics (number of true-positive, true-negative, false-positive, and false-negative observations), proportion of adequate specimens, diagnostic yield, diagnostic yield of malignancy, number of needle passes performed and follow-up data.

### Outcome measures

The outcome measures were: 1) diagnostic adequacy, defined as procurement of cytological sample that was sufficient for cytological interpretation; 2) diagnostic yield, defined as the proportion of the final cytological interpretation combined of malignant, suspicious for malignant and benign; 3) pooled sensitivity and specificity; 4) number of FNA passes required to establish a diagnosis.

### Statistical analysis

Meta-analyses for diagnostic adequacy and diagnostic yield were analyzed as weighted proportions and 95% confidence intervals (CIs) for each cohort. Findings from a random-effects model were expressed as pooled risk difference (RD) with 95% CIs. P values of <0.05 were considered to be statistically significant. Heterogeneity between studies was assessed using inconsistency index *I*^*2*^ with a cutoff of > 50%. The pooled sensitivity and specificity of two cohorts (ROSE and non-ROSE) were compared using the bivariate approach. In addition, a summary receiver operating characteristic (SROC) curve was constructed using the DerSimonian–Laird random-effects model[17] Statistical analyses were executed by Review manager5.3 (The Cochrane Collaboration, Oxford, UK) and Meta-DiSc version 1.4 statistical software (Meta-DiSc, Unit of Clinical Biostatistics team of the Ramony Cajal Hospital, Madrid, Spain).

### Assessment of methodological quality

Quality of the included studies was assessed by two review authors (D.X and S.T) independently. Cochrane Collaboration’s tool was referred to assess quality and risk of bias for the randomized trials [18]. The quality of the nonrandomized studies was evaluated by the Newcastle–Ottawa Scale (NOS) [19]. It consisted of three items: patient selection, comparability of different groups, and assessment of outcome. For the comparability between the two groups, we focused on the following variables that might affect the adequate rate and diagnostic yield: age, male proportion and size of pancreatic lesions. Diagnostic accuracy studies were evaluated using the quality assessment of diagnostic accuracy studies (QUADAS) tool [20].

## Result

### Description of included studies

**Fig 1:**
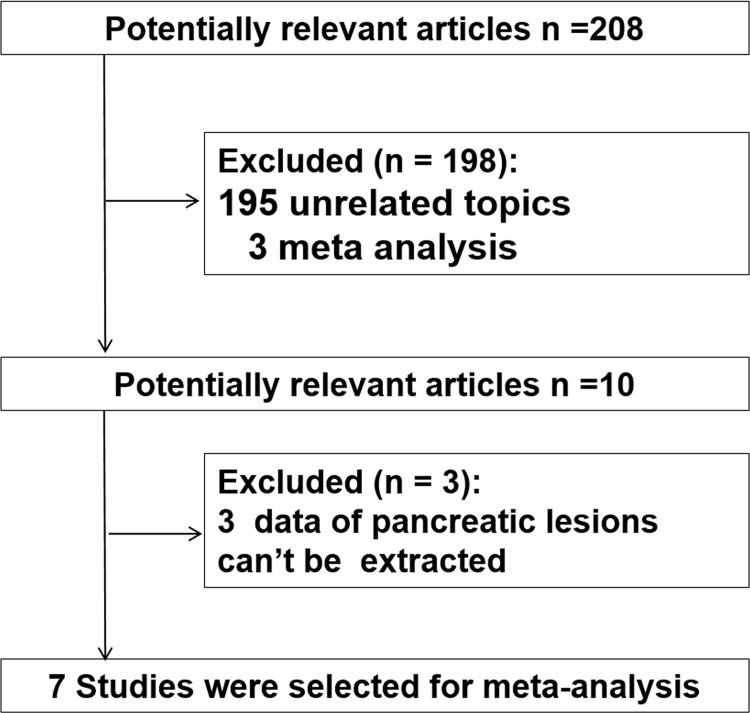
Flow chart of literature search and selection.

208 references were identified for review of title and abstract to find studies comparing EUS-FNA with and without ROSE. 198 of these were excluded because they did not fulfill the inclusion criteria. After reviewing the remaining ten manuscripts, three were subsequently excluded from this meta-analysis because they reported insufficient data for pancreatic lesions. Finally, seven comparative studies were included in this meta-analysis (Fig 1).

A total of 1299 individuals were included, in which 3 studies enrolled over 200 individuals[8,10,15], 3 studies enrolled 100–200 individuals[9,21,22], and the other one study enrolled <100 individuals[16]. Of the 7 studies, one was randomized trial [15] and the other six were non-randomized comparative studies [8–10,16,21,22]. Among the seven studies, five were focused on pancreatic lesions [8,9,15,16,21] and the other two contained detailed data for pancreatic lesions which can be extracted completely [10,22]. This analysis included five studies which performed suction during EUS-FNA [8,9,15,16,21]. Air-dried smear with Diff-Quick staining was used to perform on-site cytological evaluation in all of the included studies. Information about number of true-positive (TP), true-negative (TN), false-positive (FP) and false-negative diagnoses (FN) were recorded in four studies [9,15,16,21]. Three studies included data of follow-up information [9,15,21], of which the minimum duration of follow-up was 12 months. The patient and study characteristics are summarized in Table 1.

**Table 1:** Characteristics of patients and studies included in the meta-analysis comparing EUS-FNA with and without ROSE.

Reference	year	Country	center	design	No.	needle size, G	age, years (mean±SD)		male sex(%)		lesion size (mm)	
							ROSE+	ROSE-	ROSE+	ROSE-	ROSE+	ROSE-
**Alsohaibani**	2009	Canada	single	R	102	22	61,range(26–86)	65,range(23–84)	27(49)	23(49)	NR	NR
**eveland**	2010	USA	single	R	247	22	NR	NR	NR	NR	NR	NR
**Iglesias-Garcia,**	2011	Spain	single	R	182	22	62 range(24–84)	59 range(20–83)	57(60)	52(60)	31.7±13.6	30.7±12.9
**Cermak**	2012	USA	single	R	381	22	NR	NR	NR	NR	NR	NR
**Nayar**	2013	UK	single	R	179	22/25	63.1±17	64.4±15	48(48.5)	41(50)	32.6±23	34.7±19
**Ganc**	2015	Brazil	single	R	48	22	NR	NR	NR	NR	NR	NR
**Wani**	2015	USA	multicenter	RCT	241	22	66.1(10.2)	65.6(11.7)	74(61.2)	75(62.5)	3.3(1.4)	3.2(1.2)

R, retrospective; RCT, randomized controlled trial; NR, not reported.

### Quality assessment

The quality of each study included was assessed using different quality criteria as previous described. As to the methodological quality of the randomized trial [15], random sequence generation and adequate allocation concealment were evident and those adequately addressing incomplete outcome data were judged to be free of selective outcome reporting. The quality of nonrandomized studies were evaluated by NOS. One out of the six non-randomized studies (16.7%)[10] were ranked at level 2 (0–5) and five studies (83.3%) [8,9,16,21,22]were ranked at level 1 (6–9). Besides, all of the four studies included for accuracy assessment were classified to be high quality with a score higher than ten[9,15,16,21] (Tables 2 and 3).

**Table 2:** Outcome measures for individual studies of meta-analysis comparing EUS-FNA with and without ROSE.

Reference	Pathologist	needle Passes(mean, +SD)		Adequacy(N/Total)		diagnosis yield(N/Total)		definite for malignancy(N/Total)		Score or quality evaluation
		ROSE+	ROSE-	ROSE+	ROSE-	ROSE+	ROSE-	ROSE+	ROSE-	
**Alsohaibani**	Y	NR	NR	NR	NR	14/22	14/22	12/22	13/22	5*
**Iglesias-Garcia**	Y	2.0+0.7	3.5+1.0	94/95	76/87	92/95	67/87	76/95	43/87	6*
**Cleveland**	N	NR	NR	198/200	24/24	NR	NR	NR	NR	4*
**Cermak**	both	NR	NR	NR	NR	24/167	162/214	NR	NR	5*
**Nayar**	N	4.3+2.5	4.1+2.3	92/97	76/82	83/97	73/82	70/97	65/82	6*
**Ganc**	N	NR	NR	23/24	19/24	NR	NR	NR	NR	5*
**Wani**	Y	NR	NR	109/121	104/120	114/121	108/120	102/121	92/120	High quality^#^

ROSE, rapid on-site evaluation; NR, not reported.

^#^Randomized controlled trials (RCTs) were assessed using the Cochrane Collaboration’s tools for assessing quality.

*Non-randomized trials were assessed using the Newcastle–Ottawa Scale (NOS).

**Table 3:** Diagnostic performance for individual studies of meta-analysis comparing EUS-FNA with and without ROSE.

Reference	No.		TP		FP		FN		TN		Score
	ROSE+	ROSE-	ROSE+	ROSE-	ROSE+	ROSE-	ROSE+	ROSE-	ROSE+	ROSE-	
**Wani**	121	120	91	86	0	0	14	13	16	21	12*
**Ganc**	24	24	16	12	0	0	1	5	7	7	11*
**Nayar**	97	82	71	64	0	0	8	9	18	9	11*
**Iglesias-Garcia**	95	87	76	43	0	0	3	12	16	32	11*

ROSE, rapid on-site evaluation; TP, true positive; FP, false positive; FN, false negative; TN, true negative.

*Quality of study was evaluated using the Quality Assessment of Diagnostic Accuracy Studies (QUADAS) tool.

### Effect of ROSE on cytological adequacy

**Fig 2:**
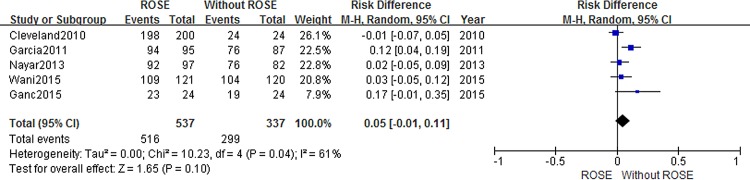
Forest plot displaying the Risk Difference and 95% CIs of each study for the adequacy rate.

**Fig 3:**
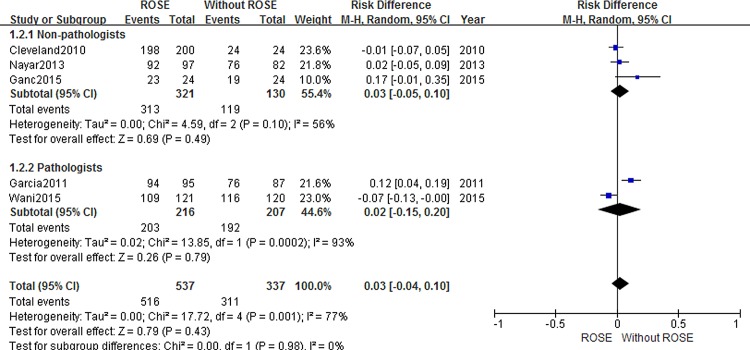
Forest plot displaying the impact of assessor type to adequacy rate.

Details of the outcomes were summarized in Table 2 and diagnostic adequacy was reported in five studies [9,10,15,16,21]. Although the cohort with ROSE service tended to have a higher adequate rate (RD = 0.05, 95% CI -0.01–0.11), this difference did not reach statistical significance. The impact of ROSE on adequacy is presented in Fig 2. However, a high heterogeneity was detected among the included studies (*I*^*2*^ = 61%). Subgroup analysis was conducted to determine whether the ROSE assessor type (cytopathologist vs nonpathologist) was a source of heterogeneity in the ROSE group (Fig 3). Conclusively, we still found no significant difference in cytological adequency. The risk difference was 0.03 (95%CI:-0.05, 0.10) and 0.02 (95%CI: -0.15, 0.20) when ROSE was performed by cytopathologist and non-cytopathologist, respectively.

### Effect of ROSE on diagnostic yield

**Fig 4:**
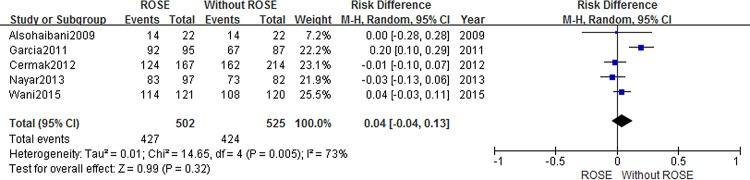
Forest plot displaying the Risk Difference and 95% CIs of each study for the diagnosis yield.

**Fig 5:**
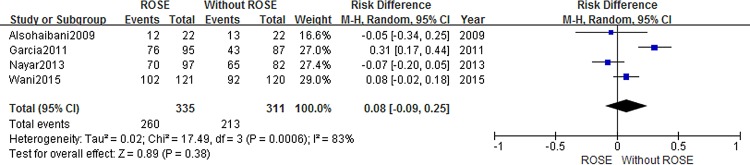
Forest plot displaying the Risk Difference and 95% CIs of each study for the diagnosis yield of malignancy.

Information about diagnostic yield was extracted from five articles [8,9,15,21,22]. Studies reporting diagnostic yield showed a 4% decrease when service of ROSE was not available (RD = 0.04 95%CI:-0.04, 0.13). But this difference between the two cohorts was not statically significant (Fig 4). To explore the impact of ROSE on diagnostic ability for malignancy, we conducted second analysis. With a combined end point of suspicious for malignancy and malignant, no statistically significant difference was found between cases with and without ROSE on diagnostic yield of malignancy (RD = 0.08 95%CI:-0.09, 0.25)(Fig 5).

### Effect of ROSE on diagnostic characteristics

There were four studies containing the intact data about diagnostic characteristics (number of true-positive, true-negative, false-positive, and false-negative observations) (Table 3) [9,15,16,21]. The between studies variability (i.e. heterogeneity) was acceptable with an *I*^2^ of 47.5% for the pooled sensitivity in the ROSE cohort.

**Fig 6:**
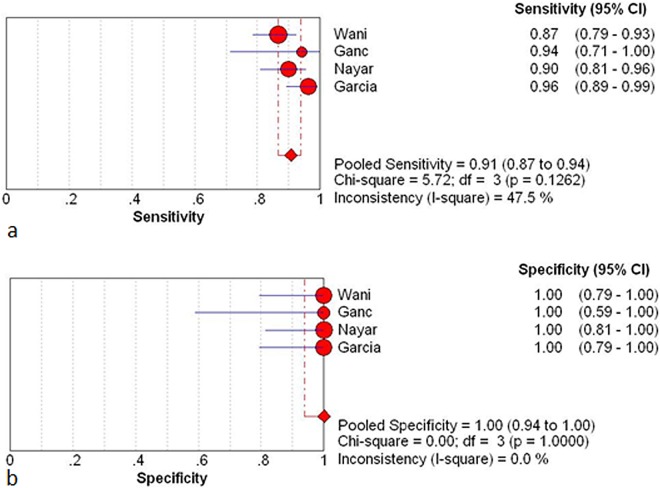
Results for the ROSE group in individual studies and from pooled data shown as forest plots for: a sensitivity; b specificity. The *I*^*2*^ result for heterogeneity is also stated (CI, confidence interval; df, degrees of freedom).

**Fig 7:**
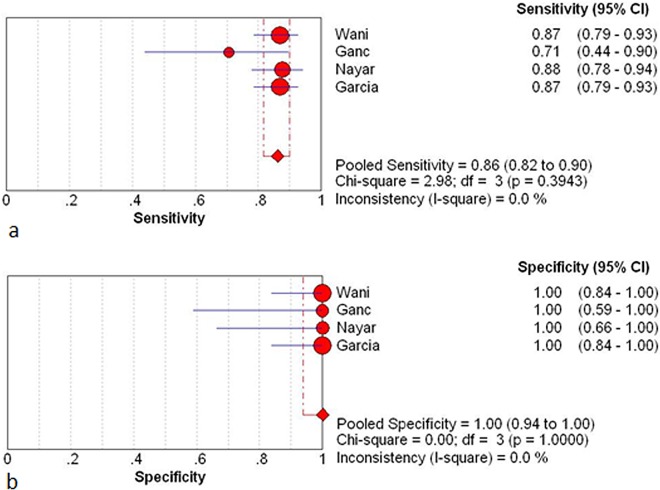
Results for the non-ROSE group in individual studies and from pooled data shown as forest plots for: **a** sensitivity; **b** specificity. The *I*^2^ result for heterogeneity is also stated (CI, confidence interval; df, degrees of freedom).

The pooled sensitivity and specificity of ROSE group were 0.91(95%CI: 0.87, 0.94) and 1 (95%CI: 0.94, 1.00) respectively. The pooled sensitivity and specificity of non-ROSE group were 0.85 (95%CI: 0.80, 0.89) and 1 (95%CI: 0.95, 1.00) (Fig 6 and Fig 7).

**Fig 8:**
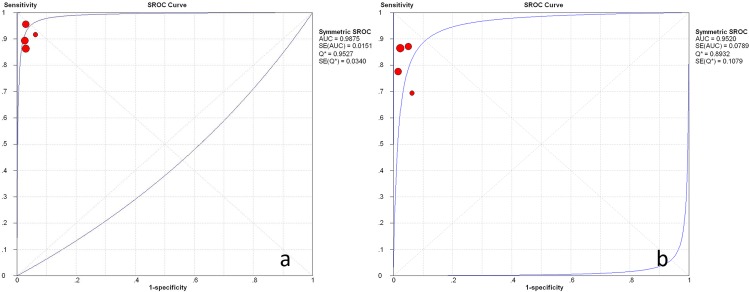
Weighted summary receiver operating characteristic (SROC) curve, with 95% confidence interval (CI), for studies involved. **a** ROSE group; **b** non-ROSE group.

The pooled positive and negative likelihood ratio of the ROSE group were 28.15 (95%:7.0, 111.88) and 0.10 (95%CI: 0.07, 0.14) respectively. The pooled positive and negative likelihood ratio for the non-ROSE group were 29.08 (95%CI: 7.44, 113.69) and 0.16 (95%CI: 0.12, 0.21) (Fig 8). The symmetric curve shows a trade-off between sensitivity and specificity. The area under the SROC was 0.99 and 0.96 for the ROSE group and non-ROSE group, which indicated high accuracy.

We analyzed the retrospective (n = 3) and prospective studies (n = 1) separately. The pooled sensitivity estimates for the ROSE group and non-ROSE group among the retrospective studies were 0.93 (95%CI: 0.89–0.96) and 0.84 (95%CI: 0.81–0.88) respectively, and in the prospective study was 0.87 (95%CI: 0.79, 0.93) and 0.87 (95%CI: 0.79, 0.93) respectively. Comparison of sensitivity between two cohorts in prospective study showed concordant result with the pooled sensitivity, while that in retrospective study had a higher tendency in the ROSE group.

### Needle passes

Endosonographers seemed not to perform less needle passes with the service of ROSE from the available evidence (RD = -0.68 95%CI:-2.35, 0.98). However, the randomized controlled trial which was excluded for the deficiency of mean value demonstrated that less required needle passes was associated with presence of on-site cytopathology evaluation (median, ROSE group 4 vs. non-ROSE group 7, P <0.0001).

## Discussion

This meta-analysis and systematic review included a large cohort of patients (n = 1299) and firstly enrolled evidence from randomized controlled trial. Available studies were quantitatively summarized to compare the diagnostic performance of EUS-FNA for pancreatic lesions with and without ROSE. Cytological material acquired by EUS-guided FNA is essential to the performance of ROSE. Cytological sample was expressed onto a glass slide after FNA and stained with Diff-Quick method in all of the included studies for cytological on-site evaluation, which is important to make the results from different studies more comparable, together with other standard sample preparation procedures.

Cellular adequate rate is one of the major concerns when EUS-FNA was performed in pancreatic masses. Obtaining an adequate cellular sample is fundamental to making an accurate diagnosis. Our results suggested that there was no statistically significant difference of diagnostic adequacy between groups of EUS-FNA with and without ROSE service. It should be noticed that out of the seven included articles, only one reported improved adequacy with the service of ROSE [9]. In the past decade, service of ROSE is generally believed to bring high adequate rate in the procedure of EUS-FNA [23–26]. However, most of evidence for pancreatic lesions were collected from single-cohort studies [27–30], which had low power to detect the impact of ROSE. A retrospective study enrolled 523 patients reported that presence of ROSE improved adequacy in lymph nodes, whereas no difference was made in pancreatic masses[31]. According to the result from the only randomized controlled trial so far, Wani et al showed that the service of ROSE is not associated with better cellular adequacy. Subgroup analysis demonstrated that assessor type was not correlated with the diagnostic adequacy in this study. Neither the presence of cytopathologist nor non-cytopathologist could provide a higher adequate rate. Result from three previous meta-analysis evaluating the diagnostic adequacy with regard to the role of ROSE are conflicting [12–14]. The most recent one showed that as ROSE had a higher success rate when performed by pathologists rather than non-pathologists, the assessor type was also correlated with the non-ROSE adequacy rate, which indicated that the success rate with ROSE may depend on the non-ROSE success [13].

The newly published randomized controlled study included in this meta-anlysis made large contribution to find the effect of ROSE on adequacy, simultaneously being the only study reported significant difference of needle passes (median, ROSE group 4 vs. ROSE− 7, P <0.0001). So, it is reasonable to make a connection that less needle passes was required with the service of ROSE. In addition, the area of puncture could be changed according to the information provided by the on-site cytopathologists in cases of inadequate samples. This may impact the number of passes needed to obtain an appropriate sample too. A prospective study by LeBlanc et al. found that at least 7 passes for pancreatic masses are needed during a EUS procedure without presence of a cytopathologist [32]. Erickson et al presented a similar result that without a cytopathologist in attendance, 5 to 6 passes should be performed for pancreatic masses [33]. Unfortunately, we found no significant difference of needle passes existing between the two cohorts in our meta-analysis. This is probably because we excluded data of the randomized controlled trial out of this comparison for its vacancy of mean value of needle passes. Lessons could be learned from this high quality study is that the additional passes with a clinical significance might improve the sampling adequacy, especially for those centers without ROSE. However, more prospective evidence are still needed to support this strategy applied in clinical centers.

The diagnostic yield of EUS-FNA with ROSE was reported higher than 90% in most studies; however, similarly good results have been reported from selected studies without ROSE. In this study, we found no evidence that implementation of ROSE improved the diagnostic yield (RD = 0.04 95%CI:-0.05, 0.13). For the sake of pre-defined diagnostic yield (malignant, suspicious for malignancy and benign), bias from definition variation was greatly reduced in our study. A meta-analysis found that evidence supporting this result was more convincing at centers with low adequacy rates (<90%)[13]. Therefore, routine use of ROSE may not change outcomes in clinical practice at tertiary care centers. It is also demonstrated in our study that using a combined end point of suspicious for malignancy and malignant results, no statistically significant difference was found in diagnostic yield of malignancy with and without ROSE (RD = 0.08 95%CI:-0.09, 0.25).

In this study, available data regarding the impact of ROSE on the diagnostic performance were also summarized. Four studies (three retrospective studies and one RCT) with complete information were included. The pooled sensitivity, specificity, likelihood ratios for a positive or negative test (LR+, LR-) and summary receiver-operation curves (SROC) were determined. The result of calculation showed that the presence of ROSE did not provide superior sensitivity and specificity. It has been demonstrated that ROSE group did not have a beneficial effect on cellular adequacy. So, the above finding deemed reasonable because similar cellular yield are supposed to result in comparable diagnostic efficacy. Second analysis was performed in retrospective and prospective studies separately. Pooled sensitivity from retrospective studies elucidated a significant rise with presence of ROSE while that from the prospective study did not. Considering the high quality of RCT, this difference is supposed to result from a design bias. Numerous studies have reported the impact of ROSE on diagnostic accuracy in a single cohort design. Based on these evidence, a recent meta-analysis suggested that higher diagnostic accuracy was associated with the presence of ROSE. However, single-cohort studies are difficult to isolate the incremental impact of ROSE from other factors that ordinarily vary from institution to institution.

EUS-FNA combined with cytological interpretation has been acknowledged to a safe and effective method providing a high diagnostic accuracy (range, 66–96%). However, repeat EUS would be advised to perform if clinically indicated and when the first EUS–FNA was non-diagnostic. Repeat procedure was not analyzed in this study for the short of available information. In a retrospective study by Collins et al enrolling 756 patients, the non-ROSE group had twice as many repeat EUS-FNA biopsies as that in the ROSE group (22 biopsies vs 11 biopsies) and the difference was statistically significant (P<0.024). Besides, the second biopsies in the ROSE cohort had a higher rate of definite diagnosis (63%) compared with the non-ROSE cohort (27%)[31]. However, in a randomized controlled trial, contrary result was reported that there was no difference between the two groups with regard to the number of repeat procedure (ROSE group 5 (4.1%) vs. non-ROSE group 4 (3.3%), P = 0.59) [15]. Because the requirement for additional EUS was variable and clinical data could be referred by a doctor to make a decision, more studies are needed to answer this question clearly.

There are several limitations to this meta-analysis. First, the number of studies that met the inclusion criteria was small (n = 7). Although we included the several recently published studies, prospective designed study was still limited (n = 1). But it is worth noting that results reported by recently published RCT indeed changed some important point of views regarding the impact of ROSE, especially in diagnostic adequacy and number of needle passes. Besides, the number of needle passes was not pre-determined in some of the included studies, and the endosonographer decided how many passes had to be performed, which may be treated as a sort of evaluation. Furthermore, as it was implemented over 5 years, the study by Cermak et al. might be subject to the bias introduced by nonconcurrent comparison. Results might vary with the promotion of echoendoscope and improvement of operating experience.

In conclusion, results of this meta-analysis that compared EUS-FNA with and without ROSE demonstrated no statistically significant difference in the diagnostic yield of malignancy and proportion of patients with adequate specimens. Diagnostic sensitivity and specificity between the two groups are also comparable. Since ROSE is time-consuming service with poor reimbursement and not available in many endoscopic centers, it should be not strongly recommended to provide a ROSE service throughout all centers performing EUS for pancreatic lesions.

## Supporting Information

S1 PRISMA Checklist (DOC)
